# Full-length soluble CD147 promotes MMP-2 expression and is a potential serological marker in detection of hepatocellular carcinoma

**DOI:** 10.1186/1479-5876-12-190

**Published:** 2014-07-04

**Authors:** Jiao Wu, Zhi-Wei Hao, You-Xu Zhao, Xiang-Min Yang, Hao Tang, Xin Zhang, Fei Song, Xiu-Xuan Sun, Bin Wang, Gang Nan, Zhi-Nan Chen, Huijie Bian

**Affiliations:** 1 Cell Engineering Research Center and Department of Cell Biology, State Key Laboratory of Cancer Biology, Fourth Military Medical University, Xi’an 710032, China

**Keywords:** Hepatocellular carcinoma, Soluble CD147, Matrix metalloproteinases, Alpha-fetoprotein, Serological marker

## Abstract

**Background:**

As a surface glycoprotein, CD147 is capable of stimulating the production of matrix metalloproteinases (MMPs) from neighboring fibroblasts. The aim of the present study is to explore the role of soluble CD147 on MMPs secretion from hepatocellular carcinoma (HCC) cells, and to investigate the diagnostic value of serum soluble CD147 in the HCC detection.

**Methods:**

We identified the form of soluble CD147 in cell culture supernate of HCC cells and serum of patients with HCC, and explored the role of soluble CD147 on MMPs secretion. Serum CD147 levels were detected by the enzyme-linked immunosorbent assay, and the value of soluble CD147 as a marker in HCC detection was analyzed.

**Results:**

Full length soluble CD147 was presented in the culture medium of HCC cells and serum of patients with HCC. The extracellular domain of soluble CD147 promoted the expression of CD147 and MMP-2 from HCC cells. Knockdown of CD147 markedly diminished the up-regulation of CD147 and MMP-2 which induced by soluble CD147. Soluble CD147 activated ERK, FAK, and PI3K/Akt pathways, leading to the up-regulation of MMP-2. The level of soluble CD147 in serum of patients with HCC was significantly elevated compared with healthy individuals (*P* < 0.001). Soluble CD147 levels were found to be associated with HCC tumor size (*P* = 0.007) and Child-Pugh grade (*P* = 0.007). Moreover, soluble CD147 showed a better performance in distinguishing HCC compared with alpha-fetoprotein.

**Conclusions:**

The extracellular domain of soluble CD147 enhances the secretion of MMP-2 from HCC cells, requiring the cooperation of membrane CD147 and activation of ERK, FAK, and PI3K/Akt signaling. The measurement of soluble CD147 may offer a useful approach in diagnosis of HCC.

## Background

Hepatocellular carcinoma (HCC) is the sixth most common malignancy and the third leading cause of cancer-related death worldwide, its incidence is increasing [1]. Interactions among stromal, inflammatory, and cancer cells create a complex, permissive microenvironment that favors HCC progression [2]. Matrix metalloproteinases (MMPs) as modulators of tumor microenvironment play crucial roles in extracellular matrix turnover, cell growth, inflammation, angiogenesis, and tumor cell migration by both proteolytic and nonproteolytic manners [3]. CD147, designated as extracellular MMP inducer (EMMPRIN), is overexpressed in tumor cells and is capable of stimulating the production of various MMPs such as MMP-1, MMP-2, MMP-3, MMP-9, and MMP-11 [4,5]. Our previous study demonstrated that CD147 in tumor cells modulates fibroblasts, as well as HCC cells themselves to produce MMP-2 and MMP-9, promoting HCC invasion and metastasis [6]. Clinical evidences showed that CD147 expression in HCC tissue is correlated with MMP-2 level and is an independent predictor of poor survival in patients with HCC [7]. Membrane-bound CD147 may interact with integrin or monocarboxylate transporter family members in signal transduction for MMP induction [8-11].

Two forms of soluble CD147 were detected in the conditioned medium of cancer cells, the full-length protein and the protein containing the extracellular domain. The full-length CD147 is released by tumor cells via microvesicle shedding, stimulating MMP expression in fibroblasts through a phospholipase A (2) and 5-lipoxygenase catalyzed pathway [12,13]. CD147 may also shed from cell membrane via an MMPs-dependent cleavage, generating soluble CD147 contains either two Ig-like domains or the N-terminal Ig-like domain [14,15]. It was demonstrated that recombinant CD147 containing extracellular portion interacts with membrane-bound CD147 in fibroblasts [16]. Currently it is well established that soluble CD147 derived from tumor cell acts in a paracrine fashion on stroma cells to stimulate the production of MMPs, which consequently contributes to tumor metastasis [17,18]. Although most MMPs in cancer tissue are produced by stromal rather than cancer cells, clinical evidences showed the tumor cell-expressed MMP-2 and MMP-9 are related with HCC progress [19,20]. However, little is known the form and the level of soluble CD147 in patients with HCC and the regulation of soluble CD147 on MMP expression in HCC cells. The diagnostic value of serum soluble CD147 for HCC has not been investigated either.

In this study, we identified a full-length soluble CD147 secreted by HCC cells and verified this form in serum of patients with HCC. We investigated the cooperation between soluble CD147 and membrane-bound CD147 and activation of the downstream pathways on secretion of MMP-2 from HCC cells. Furthermore, we examined serum soluble CD147 and MMP-2 levels in patients with HCC and evaluated the results with respect to clinical features. We showed that serum soluble CD147 may serve as a diagnostic marker for HCC, especially for HCC with negative alpha-fetoprotein (AFP) and HCC at early stage.

## Methods

### Cell culture

The human HCC cell line SMMC-7721 was obtained from the Institute of Cell Biology, Chinese Academy of Sciences (Shanghai, China). 7721-shCD147 cells (*CD147* gene is stably knocked down by shRNA) and T7721 cells (*CD147* gene is stably overexpressed) were developed based on SMMC-7721 cell line and preserved in our laboratory. All cells were cultured in RPMI 1640 medium (Gibco, NY, USA) supplemented with 10% fetal bovine serum, 100 μg/ml of penicillin, and 100 μg/ml of streptomycin.

### Acetone precipitation of soluble CD147 in conditioned medium

SMMC-7721 cells were cultured in serum-free medium for 48 h and the conditioned medium was harvested. Protein sample was placed in an acetone-compatible tube. Cold (4°C) acetone was added to the tube and mixed by vortex. The mixture was then incubated for 60 min on ice. After centrifugation for 10 min at 13,000 × *g*, the supernatant was properly disposed. The protein pellet was resuspended in cold acetone and the precipitation step was repeated. Samples were analyzed by immunoblotting with antibodies against extracellular CD147 (HAb18 antibody, prepared by our laboratory) [21] or intracellular CD147 (C-19 antibody, Santa Cruz Biotechnology, CA, USA).

### Preparation of extracellular and intracellular domains of CD147

The prokaryotically and eukaryotically expressed extracellular CD147 (named as P-CD147ECD and E-CD147ECD, respectively) were prepared as previously described [22]. The cDNA fragment encoding CD147 intracellular domain (residues 227–269) was amplified by PCR, in which *Nde* I and *Xho* I restriction enzyme sites had been added, respectively. The PCR product was digested with *Nde* I and *Xho* I and ligated into the expression vector pGEX, which produced CD147 intracellular domain with a GST tag at the N-terminus. The plasmids were transformed into *Escherichia coli* strain BL21 (DE3), and protein expression was induced with 100 mg/L isopropyl-β-D-1-thiogalactopyrano-side. After 20 h of growth, GST-CD147 intracellular domain was purified from the soluble fraction using a Glutathione Sepharose 4B- column (GE Healthcare Life Sciences, New Jersey, USA). The GST tag was removed with PreScission™ Protease (GE Healthcare Life Sciences) at 4°C overnight, and CD147 intracellular domain was purified by gel filtration with Superdex 75 column in 20 mmol/L HEPES buffer (pH 7.3). The prokaryotically expressed intracellular CD147 was named as P-CD147ICD.

### Western blot

Proteins were separated by 10% SDS-containing polyacrylamide gel electrophoresis and transferred to a polyvinylidene fluoride microporous membrane (Millipore, MA, USA). The membrane was probed with primary antibodies including HAb18, C-19 (Santa Cruz Biotechnology), anti-MMP-2 (Santa Cruz Biotechnology), anti-p-ERK1/2, anti-p-FAK, anti-p-Akt, anti-p-EGFR, anti-ERK1/2, anti-Akt, anti-EGFR (Cell Signaling Technology, Danvers, USA), anti-FAK (BD), and anti-α-tubulin antibodies (Santa Cruz Biotechnology).

### Immunoprecipitation of serum soluble CD147

Immunoprecipitation was performed to detect the soluble CD147 in serum samples of patients with HCC using the Pierce Direct Immunoprecipitation Kit (Pierce Biotechnology, Rockford, USA). The agaroseresin were incubated with HAb18 antibody or C-19 antibody for 8 h at 4°C. Subsequently, the preformed agarose-antibody complexes were incubated overnight at 4°C with serum samples. The flow-through fractions of serum samples were also reserved. After washing to remove unbound components of the sample, the antigen was recovered by dissociation from the antibody with elution buffer supplied in the kit. Samples were analyzed by immunoblotting with C-19 or HAb18 antibodies.

### RNA interference

Transfection of small interfering RNAs was performed using Lipofectamine 2000 (Invitrogen, Carlsbad, USA). siRNA targeting CD147 (5′-GTACAAGATCACTGACTCT-3′) and silencer negative control siRNA (snc-RNA) were synthesized by Shanghai GenePharma Co., Ltd, China.

### Immunofluorescence

SMMC-7721, 7721-shCD147, and T7721 cells were grown on confocal dishes for 24 h, and fixed with 4% paraformaldehyde. Cells were incubated with HAb18 antibody, followed by fluorescent staining with Alexa Fluor 488-conjugated anti-mouse IgG (Invitrogen). Cell nuclei were stained with 4′,6-diamidino-2-phenylindole (DAPI). Images were obtained with an FV1000 laser scanning confocal microscope (Olympus, Japan).

### Real-time PCR

SYBR-Green real-time RT-PCR was performed as previously described [23] using SYBR Premix EX Taq II (2 ×) (Takara, Japan) with the sequence detection system Stratagene Mx3005P (Agilent Technologies, Germany). The mRNA level of specific genes was normalized against glyceraldehyde 3-phosphate dehydrogenase (GAPDH). All primers were listed in Table 1, synthesized by Shanghai Sangon Biological Engineering Technology & Services Co., Ltd.

**Table 1:** Sequences of real-time PCR primers

**Gene**	**Primer sequences**	
	**Forward**	**Reverse**
*CD147*	5′-ACTCCTCACCTGCTCCTTGA-3′	5′-GCCTCCATGTTCAGGTTCTC-3′
*MMP-1*	5′-GAAGAATGATGGGAGGCAAGT-3′	5′-GAGGACAAACTGAGCCACATC-3′
*MMP-2*	5′-GGCAGTGCAATACCTGAACACC-3′	5′-GTCTGGGGCAGTCCAAAGAACT-3′
*MMP-3*	5′-TTTTACCCTTTTGATGGACCTG-3′	5′-GTCCCTGTTGTATCCTTTGTCC-3′
*MMP-9*	5′-TTCCCCTTCACTTTCCTGGGTA-3′	5′-CGCCACGAGGAACAAACTGTAT-3′
*MMP-13*	5′-TCTTCGGCTTAGAGGTGACTG-3′	5′-CAGAGGAGTTACATCGGACCA-3′
*GAPDH*	5′-GCACCGTCAAGGCTGAGAAC-3′	5′-TGGTGAAGACGCCAGTGGA-3′

### Gelatin zymography

SMMC-7721 cells were cultured in the serum-free medium containing 0, 0.1, 1, 10, or 100 μg/ml E-CD147ECD for 48 h. Conditioned medium was harvested and the activity of MMP-2 was assayed by gelatin zymography as described previously [23].

### Enzyme-linked immunosorbent assay (ELISA)

Serum samples and sections of liver tissues were supplied by Beijing Youan Hospital, Capital Medical University. Informed consent has been obtained and the study was conducted with the approval of the institutional ethics board of Beijing Youan Hospital of Capital Medical University. The clinical and demographic characteristics of patients with HCC were shown in Table 2. The concentrations of soluble CD147 and MMP-2 were measured by ELISA. Soluble CD147 levels were assessed using a specific Human EMMPRIN/CD147 Quantikine ELISA Kit (R&D Systems, Minneapolis, MN, USA) according to the manufacturer’s protocol. Serum MMP-2 levels were measured with human MMP-2 ELISA kits (Senxiong Biotech, Shanghai, China). The concentration in each sample well was determined by interpolation from a standard curve. Each sample was tested in duplicate.

**Table 2:** Clinical and demographic characteristics of patients with HCC

**Characteristics**	***n*** **= 62**
**Age, years**	
Mean ± SD	56.6 ± 8.6
Median	57.5
Range	40–77
**Sex, n (%)**	
Male	53 (85.5)
Female	9 (14.5)
**Hepatitis history, n (%)**	
Yes	58 (93.5)
**Hepatic cirrhosis, n (%)**	
Yes	56 (90.3)

### Immunohistochemistry

Formaldehyde-fixed and paraffin-embedded sections of liver tissue were subjected to immunohistochemistry and stained with monoclonal antibodies against CD147 or MMP-2 using the streptavidin-peroxidase staining kit (ZSGB-Bio., Beijing, China). The percentage of the positive cells were graded on a scale of 0 to 4 (0: negative, 1: 0–25%, 2: 26–50%, 3: 51–75%, 4: 76–100%). Areas that showed positivity were further quantified from 0 to 3 by the relative staining intensity (1: weak, 2: moderate, 3: intensive). The final score was obtained by multiplying the intensity and quantification measurements. Images were obtained with an inverted microscope (CKX41; Olympus) equipped with a digital camera under 400× magnification.

### Statistical analysis

Mann–Whitney test or Kruskal-Wallis test was used to compare levels of serum CD147 or MMP-2 in different groups with variable clinicopathologic features. Logistic regression was used to evaluate the association between soluble CD147 and HCC risk. A Spearman’s correlation test was used to assess the correlation of serum CD147 levels with CD147 or MMP-2 levels in HCC tissues. Receiver operating characteristic (ROC) curve analysis was done to calculate area under the curve (AUC) and to identify cutoff points. AUC values and 95% confidence intervals (CI) were used to summarize diagnostic performance of CD147 and AFP. Unless otherwise stated, the results were expressed as mean ± standard deviation. One-way ANOVA test and *t*-test were performed for other experiments to compare the mean values. The *P* values and data were obtained from three independent experiments. Statistical significance was set at *P* < 0.05. Adobe Photoshop CS6 was used to quantify the intensity of bands on western blot. The GraphPad Prism software and SPSS 17.0 software were used for statistical analysis.

## Results

### Full-length soluble CD147 was presented in conditioned medium of HCC cells

**Figure 1:**
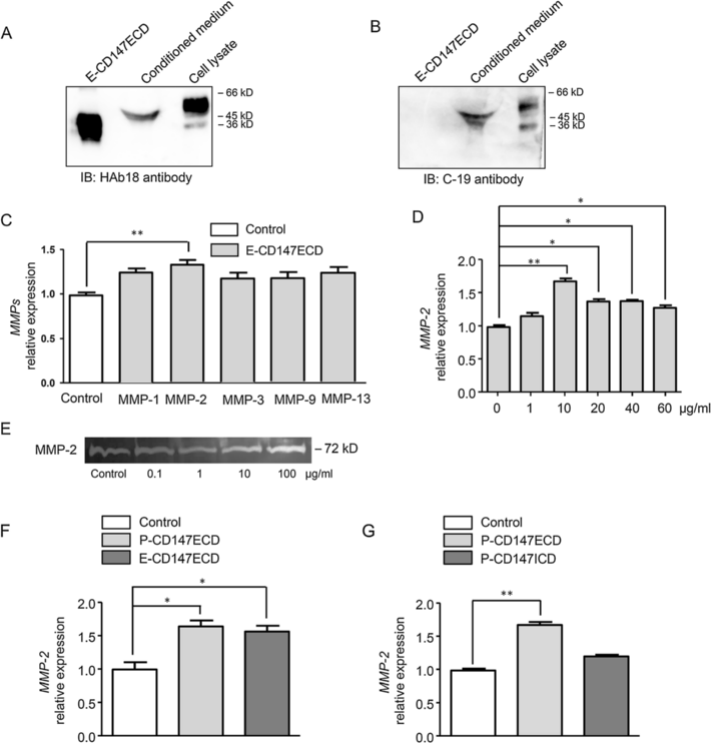
**MMPs expression in HCC cells under the stimulation of soluble CD147.** Eukaryotically expressed extracellular CD147 (E-CD147ECD), serum-free conditioned medium, and SMMC-7721 cell lysates were subjected to western blotting. HAb18 reacting against extracellular CD147 **(A)** and C-19 reacting against intracellular CD147 **(B)** were used respectively. **(C)** Real-time PCR was performed to test mRNA levels of MMP-1, -2, -3, -9, and MMP-13 in SMMC-7721 cells in the absence or presence of 10 μg/ml E-CD147ECD for 6 h. **(D)** Soluble CD147 increased the expression of MMP-2 in a concentration-dependent manner. SMMC-7721 cells were treated with different concentrations of E-CD147ECD for 6 h and MMP-2 mRNA level was analyzed using real-time PCR. **(E)** SMMC-7721 cells were treated with different concentrations of E-CD147ECD and MMPs levels were analyzed using gelatin zymography. **(F)** SMMC-7721 cells were treated with prokaryotically expressed extracellular CD147 (P-CD147ECD) or E-CD147ECD for 6 h and MMP-2 mRNA level was analyzed using real-time PCR. GAPDH was used as a normalization control. *P < 0.05. **(G)** Real-time PCR showed mRNA level of MMP-2 in SMMC-7721 cells treated with P-CD147ECD or prokaryotically expressed intracellular CD147 (P-CD147ICD) for 6 h. GAPDH was used as a normalization control in all real-time PCR analysis. *P < 0.05, **P < 0.01.

The soluble form of CD147 has been detected in conditioned medium of tumor cells as either a full-length protein, a protein lacking the transmembrane and cytoplasmic domains, or a segment only containing the N-terminal Ig-like domain [13,15]. Here we tried to identify the form of soluble CD147 from HCC cells. The serum-free conditioned medium and SMMC-7721 cell lysates were subjected to western blotting for two types of CD147 antibodies. One is HAb18 antibody reacting against extracellular domain of CD147, and the other is commercial C-19 antibody reacting against intracellular domain of CD147. The immunoblot analysis using HAb18 antibody confirmed the presence of soluble CD147 in the conditioned medium of HCC cells, yielding a band with molecular weight between 45–66 kDa (Figure 1A). While C-19 antibody also detected CD147 in the conditioned medium (Figure 1B), demonstrating the presence of intracellular domain of soluble CD147. Meanwhile, the eukaryotically expressed extracellular domain of CD147 (E-CD147ECD) [22] showed a 35 kDa-band by HAb18 antibody, and were undetected by C-19 antibody (Figure 1A and B). We concluded that a full-length soluble CD147 was present in the conditioned medium of HCC cells.

### MMP-2 upregulation mediated by extracellular domain of soluble CD147 core protein

To evaluate the effect of soluble CD147 on expression of MMPs in HCC cells, we tested the various types of MMPs, including MMP-1, -2, -3, -9, and MMP-13 in SMMC-7721 cells. Real-time PCR revealed a significant upregulation of MMP-2 under the stimulation of E-CD147ECD for 6 h (Figure 1C). We then evaluated MMP-2 mRNA level in response to different doses of E-CD147ECD in SMMC-7721 cells. The E-CD147ECD increased MMP-2 expression in a concentration-dependent manner, and 10 μg/ml CD147 had the greatest effect on stimulation of MMP-2 mRNA expression (*P* < 0.01) (Figure 1D). The secretion of MMP-2 in supernatant was also increased with recombinant CD147 stimulation as demonstrated by SDS-polyacrylamide gelatin zymography (Figure 1E). Next, HCC cells were treated with E-CD147ECD and prokaryotically expressed extracellular domain of CD147 (P-CD147ECD) for 6 h, respectively. We found that both prokaryotically and eukaryotically expressed extracellular CD147 promoted the MMP-2 expression with a similar efficiency, indicating the core protein but not the glycosylation plays the functional role (Figure 1F). In order to validate the role of extracellular domain of soluble CD147 in mediating the upregulation of MMP-2 expression, P-CD147ECD and prokaryotically expressed intracellular CD147 (P-CD147ICD) were used. As shown in Figure 1G, intracellular CD147 did not significantly up-regulated MMP-2 expression, further demonstrating the regulation of MMP-2 was mediated by the extracellular domain of soluble CD147.

### Efficient induction of MMP-2 by extracellular domain of soluble CD147 required cooperation with membrane-bound CD147

**Figure 2:**
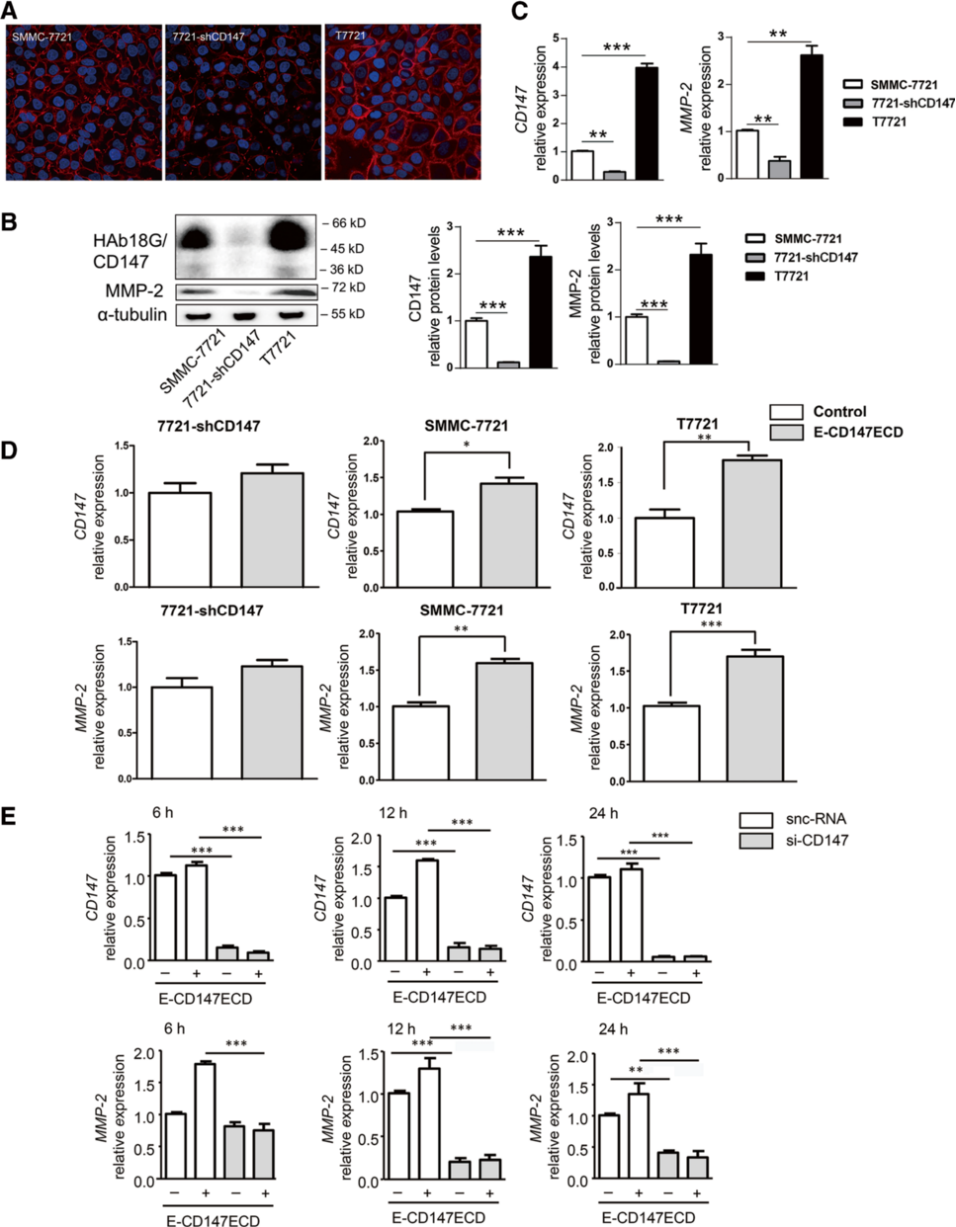
**Cooperation of soluble CD147 and membrane-bound CD147 in the induction of MMP-2 in HCC cells. (A)** Immunofluorescence of membrane-bound CD147 in SMMC-7721 cells, 7721-shCD147 cells, and T7721 cells detected by confocal laser scanning microscopy (×600). **(B)** The protein levels of CD147 and MMP-2 were analyzed by western blotting. Blots were probed for α-tubulin to ensure equal protein loading. Western blot scanning densitometry for three independent experiments. **(C)** The mRNA levels of CD147 and MMP-2 were analyzed by real-time PCR. **(D)** SMMC-7721 cells, 7721-shCD147 cells, and T7721 cells were treated with E-CD147ECD for 6 h and analyzed for CD147 and MMP-2 mRNA expression using real-time PCR. **(E)** Real-time RT-PCR detection of CD147 and MMP-2 mRNA levels in SMMC-7721 cells transfected with si-CD147 followed by stimulation with 10 μg/ml E-CD147ECD at different time points. Cells were transfected with snc-RNA as a control. GAPDH was used as a normalization control. *P < 0.05, **P < 0.01, ***P < 0.001.

To detect whether the membrane-bound CD147 was correlated with induction of MMP-2 upon the stimulant soluble CD147ECD, we generated two cell lines by stable transfection of the CD147 small hairpin RNA (shCD147) and human CD147 cDNA into SMMC-7721 cells, named 7721-shCD147 and T7721, respectively. Immunofluorescence assay demonstrated that shCD147 revealed a significant inhibition of membrane-bound CD147 levels, while location of CD147 on cell membrane was increased with CD147 cDNA transfection (Figure 2A). Western blotting analysis of cell lysates showed that a distinct loss of MMP-2 expression in 7721-shCD147 cells and a significant increase of MMP-2 level in T7721cells (Figure 2B), which was consistent with the results of real-time PCR (Figure 2C). These results suggest that membrane-bound CD147 is involved in the MMP-2 production. When the three cell lines were treated with 10 μg/ml of E-CD147ECD, real-time PCR indicated that CD147 was autocrine-upregulated with 1.20-, 1.42-, and 1.82-folds in 7721-shCD147, SMMC-7721, and T7721 cells, and MMP-2 expression was enhanced with 1.20-, 1.63-, and 1.78-folds (Figure 2D). The elevated tendency of both MMP-2 and CD147 expression was appropriated with the membrane-bound CD147 levels in the three cell lines after soluble CD147 stimulation. The CD147-targeted siRNA (si-CD147) led to a marked reduction of CD147 mRNA level in SMMC-7721 cells during 24 h, as compared with snc-RNA (*P <* 0.001). When the cells were stimulated with E-CD147ECD, siCD147 significantly inhibited the upregulation of MMP-2 mRNA level, and CD147 expression as well (*P <* 0.001) (Figure 2E). These results indicate that membrane CD147 plays an important role in the regulation of soluble CD147 on MMP-2 expression.

### Involvement of ERK, FAK, and PI3K/Akt pathways in the regulation of MMP-2 expression by soluble CD147

**Figure 3:**
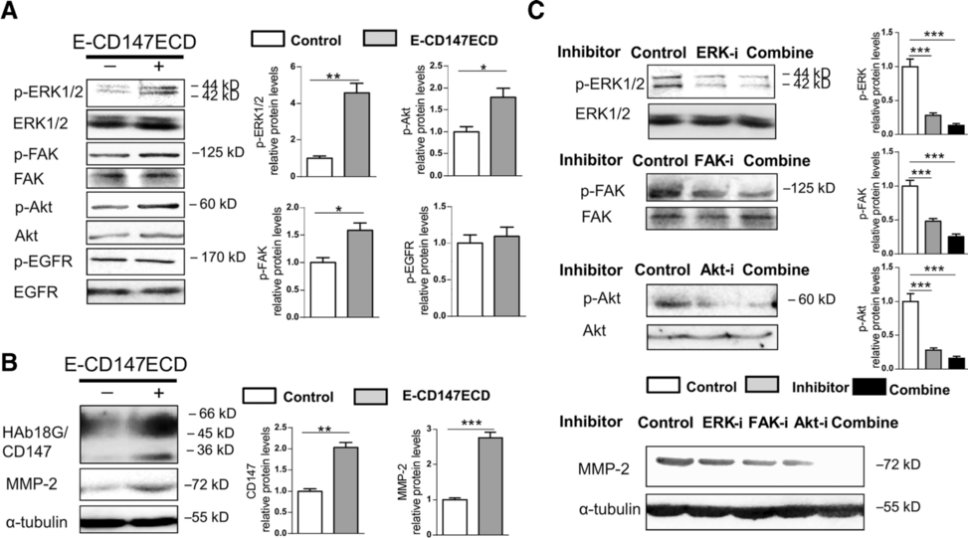
**Involvement of ERK, FAK, and PI3K/Akt pathways in upregulation of MMP-2 driven by soluble CD147.** SMMC-7721 cells were cultured for 30 min **(A)** and 12 h **(B)** with 10 μg/ml of E-CD147ECD. CD147 and MMP-2 data were expressed as means ± SEM of three independent determinations of CD147 or MMP-2/α-tubulin ratio. **(C)** SMMC-7721 cells were treated with E-CD147ECD (10 μg/ml) for 12 h with or without pre-treatment with ERK inhibitor (U0126), FAK inhibitor (FAK inhibitor 14), PI3K inhibitor (LY294002), or combined inhibitors. In A and C, the expression levels of phospho-ERK1/2, ERK1/2, phospho-FAK, FAK, phospho-Akt, Akt, phospho-EGFR, and EGFR were detected by western blotting. Quantitative analysis of phosphorylated fraction relative to the total fraction was shown. Mean values for control groups were normalized to 1. *P < 0.05, **P < 0.01, ***P < 0.001.

To define the possible regulatory role of CD147 downstream pathways on MMP-2 level, the SMMC-7721 cells were cultured with E-CD147ECD at the concentration 10 μg/ml for 30 min followed by detection of molecular phosphorylation. As shown in Figure 3A, E-CD147ECD increased the phospho-ERK1/2, phospho-FAK, and phospho-Akt contents but not phospho-EGFR content. The protein levels of CD147 and MMP-2 were elevated at 12 h post treatment (Figure 3B). In order to confirm the involvement of ERK, FAK, and PI3K/Akt signalings in upregulating MMP-2, cells were pre-treated with inhibitors U0126, FAK inhibitor 14, LY294002 (Cell Signaling Technology, Danvers, USA), or the three combination followed by incubation with E-CD147ECD for 12 h. As seen in Figure 3C, the increase in protein abundance for MMP-2 elicited by soluble CD147 was inhibited in the presence of each inhibitor, whereas the presence of all three inhibitors completely suppressed the MMP-2 increase. Taken together, these findings demonstrate that ERK, FAK, and PI3K/Akt pathways are all involved in soluble CD147-induced upregulation of MMP-2.

### Full-length soluble CD147 and secreted MMP-2 levels were elevated in patients with HCC

**Figure 4:**
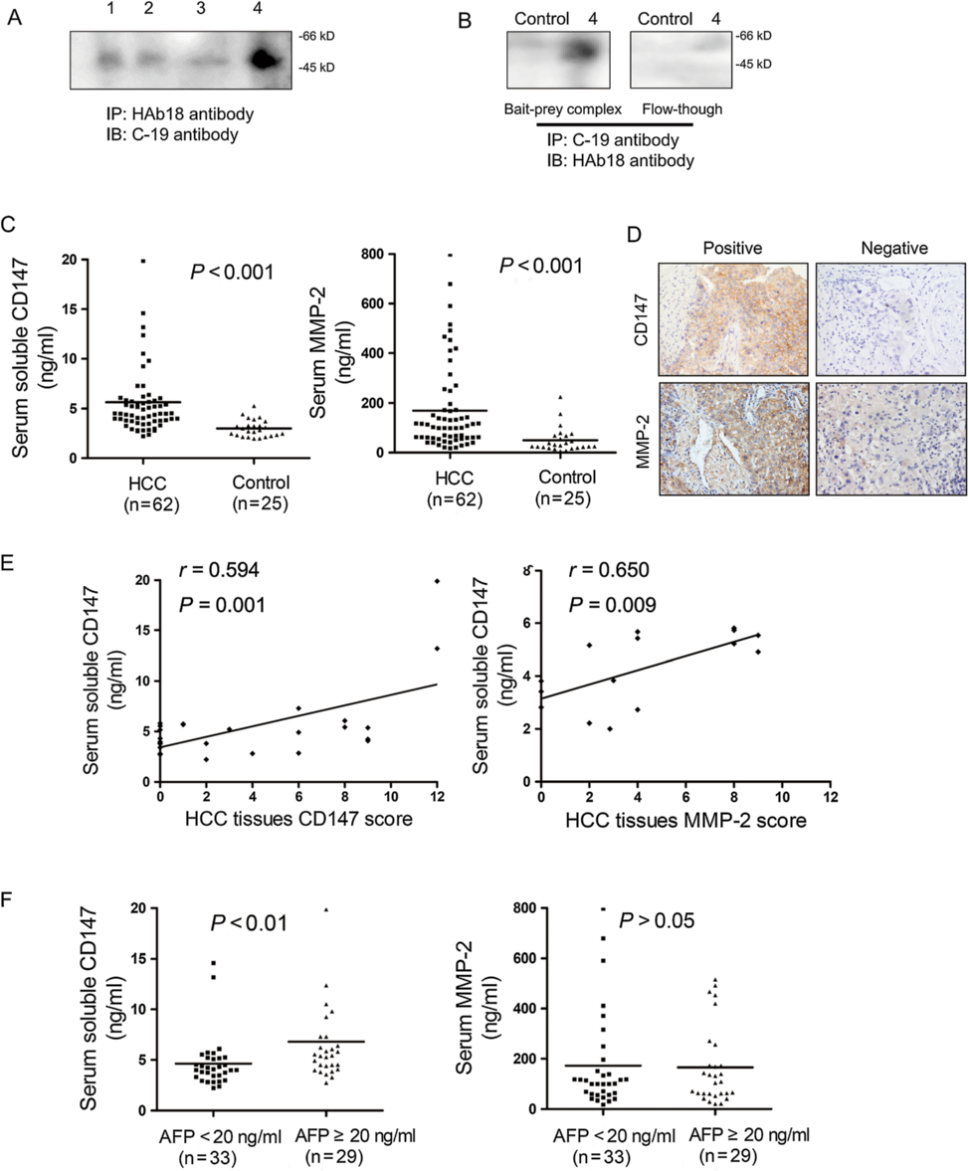
**Soluble CD147 and MMP-2 levels in the serum of HCC patients and healthy individuals. (A)** Serum samples of four HCC patients were immunoprecipitated for CD147 extracellular domain by HAb18 and immunoblotted for CD147 intracellular domain by C-19 antibody. **(B)** Serum samples of a healthy individual and number 4 patient with HCC were immunoprecipitated for CD147 intracellular domain by C-19 antibody, and the samples containing bait:prey complexes and the flow-through fractions were detected with HAb18 antibody, respectively. **(C)** Serum levels of soluble CD147 and MMP-2 were determined by ELISA assay. Bars indicated mean values in each group. **(D)** Expression of CD147 and MMP-2 in human HCC tissues assessed by immunohistochemistry (×400). Representative positive and negative samples were shown. **(E)** Correlation of serum soluble CD147 level with membrane-bound CD147 (*n* = 26) and MMP-2 expression (*n* = 15) in HCC tissues. **(F)** Comparison of serum soluble CD147 and serum MMP-2 in HCC patients with different AFP levels. Serum AFP levels were examined by electrochemiluminescence analyzer in Xijing Hospital of Fourth Military Medical University. Bars indicated mean values in each group.

To evaluate the release of soluble CD147 in blood, we tested the serum of four HCC patients. Immunoprecipitation was performed with HAb18 antibody followed by incubation with C-19 antibody. All the four HCC patients were detected the full-length soluble CD147 in serum by variation levels (Figure 4A). Furthermore, immunoprecipitation was performed with C-19 antibody, and the samples containing bait:prey complexes and the flow-through fractions were detected with HAb18 antibody by western blotting, respectively. As shown in Figure 4B, the full-length soluble CD147 was detected in the bait:prey complex from number 4 patient. However, a very weak level of soluble CD147 was detected in the bait:prey complex from healthy individual. The flow-through fractions were concentrated up to four-fold and then analyzed by western blot. A much lower level of full-length soluble CD147 was observed in the concentrated flow-through fraction from number 4 patient, and no extracellular domain of CD147 was detected. These results implied that full-length CD147 constituted the majority of soluble CD147 in serum of HCC patients. Serum soluble CD147 and secreted MMP-2 were then examined by ELISA in 62 patients with HCC and 25 healthy individuals. As shown in Figure 4C, serum soluble CD147 and secreted MMP-2 levels were significantly higher in HCC patients than in healthy individuals (*P* < 0.001) (CD147: HCC, median 4.483 ng/ml, range 2.218–20.820 ng/ml; healthy, median 2.811 ng/ml, range 1.956–5.260 ng/ml. MMP-2: HCC, median 105.7 ng/ml, range 18.2–797.5; healthy, median 28.6 ng/ml, range 5.5–224.2 ng/ml). However, serum soluble CD147 showed no significant correlation with secreted MMP-2 levels in patients with HCC (Additional file 1: Figure S1). Logistic regression model was further used to analyze the association of soluble CD147 level with HCC risk by estimating odds ratio (OR). Receiver operating characteristic (ROC) curve analysis was done to evaluate the optimal cutoff point, which was given by the maximum of the Youden index. In logistic regression analysis, soluble CD147 greater than the cutoff (3.3 ng/ml) had increased HCC risk (OR = 16.467, 95% CI: 5.264–51.507, *P* < 0.001).

### Positive correlation of soluble CD147 with membrane-bound CD147 and MMP-2 in patients with HCC

We collected 26 pairs of serum and HCC biopsy tissues. Immunohistochemistry of HCC tissues revealed the location of CD147 on cell membrane within tumor compartment and MMP-2 expression in both tumoral and stromal compartments (Figure 4D). In total, 16 of 26 HCC cases (61.5%) showed overexpression of CD147 and 12 of 15 HCC cases (80.0%) indicated MMP-2 overexpression. The score of membrane-bound CD147 expression in tumors was positively correlated with the concentration of soluble CD147 in serum (*r* = 0.594, *P* = 0.001) (Figure 4E). MMP-2 level in tumor loci was scored and showed a positive correlation with the concentration of soluble CD147 in serum (*r* = 0.650, *P* = 0.009) (Figure 4E).

### Positive correlation of soluble CD147 with serum AFP level in patients with HCC

The correlation of soluble CD147 level and secreted MMP-2 with AFP level was evaluated, respectively. As shown in Figure 4F, soluble CD147 level was significantly higher in serum of patients with AFP level over 20 ng/ml than in patients with lower AFP level (*P* < 0.01). However, no statistical significance was observed between MMP-2 levels in serum of patients with different AFP levels.

### Correlation of serum soluble CD147 with clinicopathologic features of HCC patients

The levels of soluble CD147 correlated with variable clinicopathologic factors were summarized in Table 3. The CD147 concentration was elevated in patients with Child-Pugh grade B compared with that with Child-Pugh grade A (*P* = 0.007). Elevation of CD147 concentrations was found with increased tumor size (*P* = 0.007). It was also significantly higher in patients with advanced HCC (BCLC stage C) as compared with very early HCC (BCLC stage 0) (*P* = 0.044). As shown in Table 4, serum MMP-2 levels showed no significant correlation with clinicopathologic features of HCC patients.

**Table 3:** Relationship between soluble CD147 and variable clinicopathologic features

**Clinicopathologic features**	***n*** **= 50**	**Soluble CD147**		***P *** **value**
		**Range (ng/ml)**	**Median (ng/ml)**	
**Child-Pugh grade**				0.007
A	37	2.218–19.890	4.308	
B	13	3.306–20.820	5.727	
**Tumor number**				0.470
Single	28	2.727–19.890	4.427	
Multiple	22	2.218–20.820	5.232	
**Tumor size (cm)**				0.007
<3	24	2.818–6.382	4.301	
3–5	17	2.218–20.820	4.308	
≥5	9	4.484–12.370	5.845	
**BCLC stage**				0.195
0	12	2.973–6.220	3.991	
A	19	2.727–19.890	4.482	
B	13	2.218–20.820	4.909	
C^#^	6	3.745–12.370	6.359	

^#^Significant difference as compared with BCLC 0 group (*P* = 0.044). BCLC denotes Barcelona Clinic Liver Cancer Staging System.

**Table 4:** Relationship between serum MMP-2 and variable clinicopathologic features

**Clinicopathologic features**	***n*** **= 50**	**Serum MMP-2**		***P *** **value**
		**Range (ng/ml)**	**Median (ng/ml)**	
**Child-Pugh grade**				0.401
A	37	18.2–679.1	108.1	
B	13	28.7–316.1	98.7	
**Tumor number**				0.899
Single	28	18.2–679.1	104.3	
Multiple	22	20.4–590.6	99.3	
**Tumor size (cm)**				0.284
<3	24	18.2–679.1	115.5	
3–5	17	20.4–419.3	70.2	
≥5	9	28.7–172.7	62.5	
**BCLC stage**				0.270
0	12	18.2–679.1	113.1	
A	19	40.3–590.6	113.0	
B	13	20.4–168.1	61.4	
C	6	40.4–411.0	101.9	

### Better diagnostic performance of serum CD147 compared with AFP in HCC

**Figure 5:**
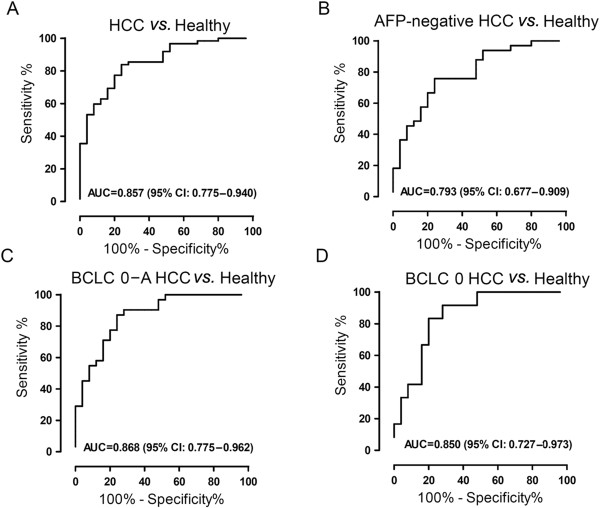
**ROC curves used to predict performance of soluble CD147 for distinguishing HCC from healthy controls. (A)** ROC curve evaluating those with HCC (*n* = 62) and healthy controls (*n* = 25). **(B)** ROC curve evaluating those with AFP-negative HCC (*n* = 33) and healthy controls (*n* = 25). **(C)** ROC curve evaluating those with early stage HCC (*n* = 31) and healthy controls (*n* = 25). **(D)** ROC curve evaluating those with very early stage HCC (*n* = 12) and healthy controls (*n* = 25).

We analyzed the ROC curves to evaluate the efficacy of serum CD147 as a diagnostic biomarker for distinguishing HCC patients from healthy individuals (Figure 5A). AUC for serum CD147 was 0.857, indicating excellent discrimination (95% CI: 0.775–0.940). Although AUC for serum CD147 was found to be larger than that of serum AFP (0.813; 95% CI: 0.726–0.900, Additional file 2: Figure S2), no statistical significance was observed. Optimal cutoff point of serum CD147 was 3.3 ng/ml, which provided a sensitivity of 83.9% (52 of 62 HCC patients correctly classified) and a specificity of 76.0% (19 of 25 controls correctly classified). The currently recommended clinical cutoff value (20 ng/ml) was used for AFP, and the sensitivity of AFP (48.4%) was much lower than that of serum CD147. We also noticed an acceptable discrimination of serum CD147 for distinguishing AFP-negative (<20 ng/ml) HCC patients from healthy individuals (AUC, 0.793; 95% CI: 0.677–0.909) (Figure 5B). Based on the optimal cutoff value of 3.3 ng/ml, 25 of 33 AFP-negative HCC patients were correctly classified (75.8%). We next focused on the subset of patients with early stage HCC (BCLC 0–A). Serum CD147 had an excellent performance for distinguishing early stage HCC (BCLC A) as well as very early stage HCC (BCLC 0) from healthy controls (Figure 5C, D). In detecting HCC of BCLC 0–A stage, the sensitivity of serum CD147 was much higher than that of AFP (87.1% vs. 45.2%, Table 5). Even in HCC with BCLC 0 stage, serum CD147 showed higher sensitivity than that of AFP (83.3% vs. 41.7%). These results suggest that serum soluble CD147 is a more sensitive marker superior to serum AFP in detecting HCC, including HCC at early stage.

**Table 5:** Sensitivity of serum CD147 and AFP in HCC patients according to BCLC stage

**Serological marker**	**Cut off value (ng/ml)**	**BCLC stage**	
		**0 n (%)**	**0**–**A n (%)**
Serum CD147	3.3	10 (83.3)	27 (87.1)
AFP	20	5 (41.7)	14 (45.2)
Combined	3.3; 20	11 (91.7)	29 (93.5)

## Discussion

CD147 is found to be overexpressed in many cancers, suggesting that it serves as a key regulator for oncogenesis and cancer metastasis [23,24]. In addition to the expression as a membrane-bound form, a soluble form of CD147 has been detected in cultured supernatants of cancer cells and human body fluids [13,17,18,25-29]. Although both the full-length soluble CD147 and the extracellular domain of CD147 in the conditioned medium of several types of cancer cells had been detected, which kind of forms existed in the serum of cancer patients was still unkown. In this study, we found the secretion of intact CD147 from HCC cells and verified this presence in the serum of patients with HCC. Over-expression of *CD147* gene into human breast cancer cells elevated soluble CD147 level, indicating the soluble CD147 release is correlated with the degree of CD147 expression in tumor cells [17]. In our study, the concentration of soluble CD147 in serum was positively correlated with CD147 expression level in HCC tissues, which was consistent with the findings *in vitro* and suggests that CD147 was secreted from tumor cells, possibly by vesicle shedding.

The homophilic interaction between soluble CD147 and its cognate receptor in cells has been demonstrated to stimulate the expression of MMPs in fibroblasts and tumor cells [16]. We showed that only the extracellular domain of soluble CD147 core protein was indispensable for the MMP-2 inducing activity, demonstrating the essential role of this domain in homophilic interactions. By gain- and loss-of-function strategies, the induction of MMP-2 by soluble CD147 was showed dependent with expression of cell associated-CD147. We also observed that soluble CD147 stimulated the upregulation of cell associated-CD147, displaying an autocrine CD147 feedback loop in HCC cells. The similar result was reported previously in primary normal human fibroblasts of lung by other group [17].

Purified recombinant CD147 protein activates multiple transcription factors in cardiomyocytes and induces interleukin-18 expression via Rac1-dependent PI3K/Akt/IKK/NF-κB and MKK7/JNK/AP-1 signaling [30]. Our previous studies had proven the production of MMPs from HCC cells induced by recombinant CD147, and the activation of ERK signaling might be involved [31]. Here, by incubating HCC cells with recombinant extracellular CD147, we observed increased phosphorylation of ERK1/2, FAK, and Akt, but not that of EGFR. A combined treatment of inhibitors targeting these pathways completely inhibited expression of MMP-2, demonstrating the cooperation of ERK, FAK, and PI3K/Akt pathways in soluble CD147-regulated MMP-2 expression. Previous study in our lab had showed a crucial role of membrane-bound CD147-integrin interaction in activation of FAK-PI3K pathway for MMPs expression [8,10]. It is reasonable to presume FAK signaling and PI3K/Akt signaling as potential pathways involved in soluble CD147-induced MMP-2 expression through membrane-bound CD147. It is worth to note other receptors may exist for soluble CD147 in ERK1/2 signaling transduction [16].

To determine relative mass values for naturally occurring soluble CD147, we used the Quantikine Human EMMPRIN kit produced by R&D Systems, Inc. In contrast to a previous study [26], we found significantly higher soluble CD147 levels in HCC patients than in healthy individuals. This results leads to the suggestion that the measurement of serum soluble CD147 may offer a useful approach in the early diagnosis and risk stratification of HCC. Although MMP-2 levels in patients with HCC were also found to be significantly higher than that in controls, there was no significant correlation between serum MMP-2 and serum soluble CD147. A previous study showed that serum MMP-2 levels in HCC patients are comparable to that in patients with chronic liver disease without this malignancy, indicating MMP-2 may not be used as a serologic marker for HCC [32].

AFP is the most utilized diagnostic serum marker for HCC [33]. However, nonspecific elevation was found in patients with liver cirrhosis and chronic hepatitis. In addition, normal AFP levels are present in many patients with early stage HCC, thus the accuracy of serum AFP in early diagnosis of HCC was not satisfying [34]. In our study, serum soluble CD147 showed significant advantages to distinguish HCC compared with AFP, including early HCC and HCC with normal serum AFP levels. The combination of soluble CD147 and AFP further improved the detective sensitivity of early stage HCC, implying a promising strategy for early diagnosis of HCC.

## Conclusions

Although membrane CD147 has been proven to be an independent predictor of poor survival in HCC patients, little is known of the diagnostic value of soluble CD147 in HCC detection. Here we found extracellular domain of soluble CD147 enhances the secretion of MMP-2 from HCC cells, requiring the cooperation of membrane CD147 and activation of ERK, FAK, and PI3K/Akt signaling. Serum soluble CD147 was elevated in HCC patients compared with healthy individuals and was associated with tumor size and Child-Pugh grade. Moreover, serum soluble CD147 showed a better performance in distinguishing HCC compared with AFP. Our study supports the use of soluble CD147 as a diagnostic marker in HCC detection, especially for HCC with negative AFP and HCC at early stage. Identification of this potential serologic marker may help to present the best chance for curative treatment and to improve outcomes. The study also provides a new insight into the role of soluble CD147 in MMP-2 secretion and HCC progression.

## Abbreviations

MMPs: matrix metalloproteinases; HCC: hepatocellular carcinoma; EMMPRIN: extracellular MMP inducer; AFP: alpha-fetoprotein; ELISA: enzyme-linked immunosorbent assay; ROC curve: receiver operating characteristic curve; AUC: area under the curve.

## Competing interests

The authors have declared that no competing interest exists.

## Authors’ contributions

WJ, HZW and ZYX carried out cell culture, western blotting analysis, real-time PCR analysis, immunofluorescence, gelatin zymography enzyme-linked immunosorbent assay. YXM, TH and ZX prepared extracellular and intracellular domains of CD147. SXX, WB and NG carried out immunohistochemistry. ZNC, HB and WJ supervised the project, participated in the design of the study and performed the statistical analysis. All authors read and approved the final manuscript.

## Supplementary Material

Additional file 1: Figure S1 Correlation analysis between serum levels of MMP-2 with serum levels of soluble CD147. Click here for file

Additional file 2: Figure S2 ROC curve of AFP evaluating those with HCC (*n* = 62) and healthy controls (*n* = 25). Click here for file
