# Supplementary figures and images for: Full-length soluble CD147 promotes MMP-2 expression and is a potential serological marker in detection of hepatocellular carcinoma
Source: J Transl Med. 2014 Jul 4;12:190. doi: 10.1186/1479-5876-12-190 (PMC4227008; doi:10.1186/1479-5876-12-190)

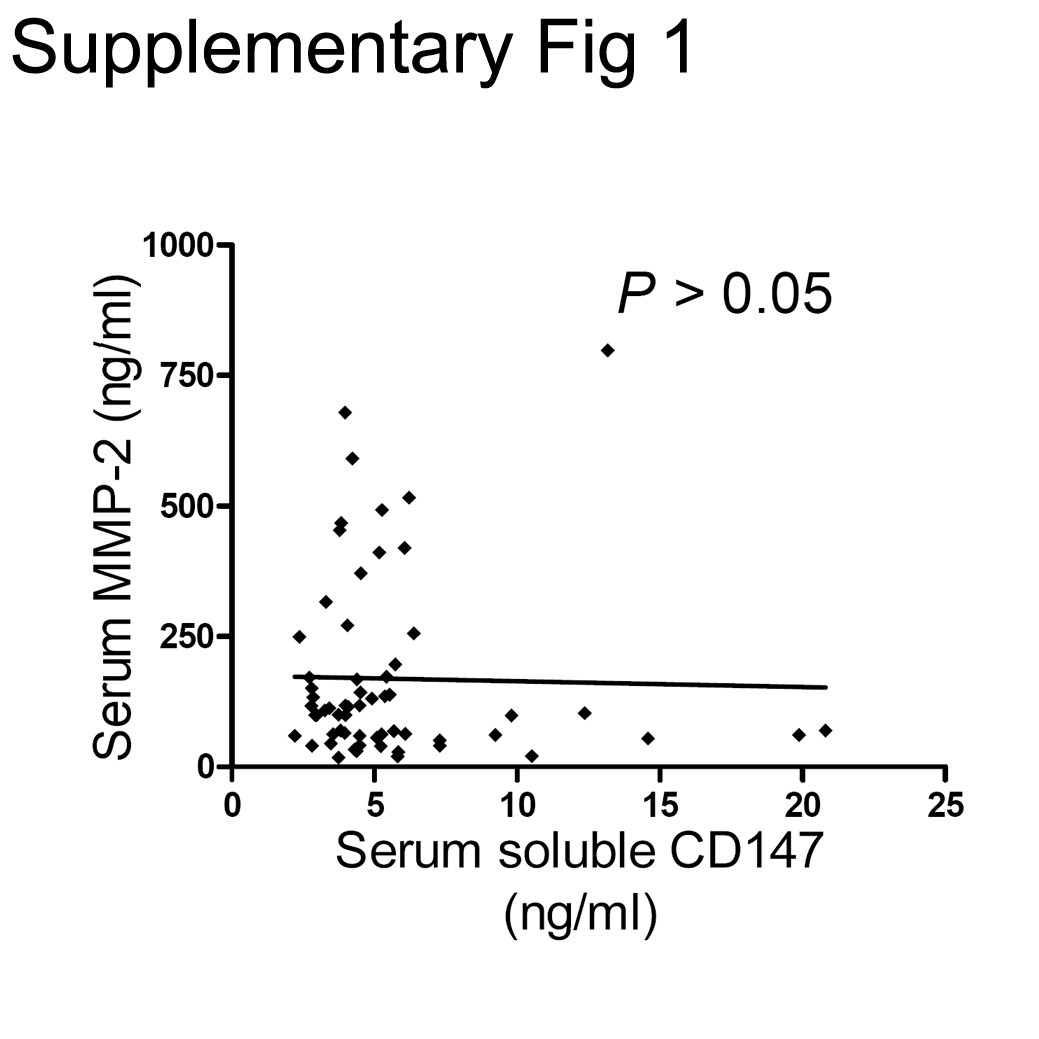

Supplement: Additional file 1: Figure S1 — Correlation analysis between serum levels of MMP-2 with serum levels of soluble CD147. [file 1479-5876-12-190-S1.tif]

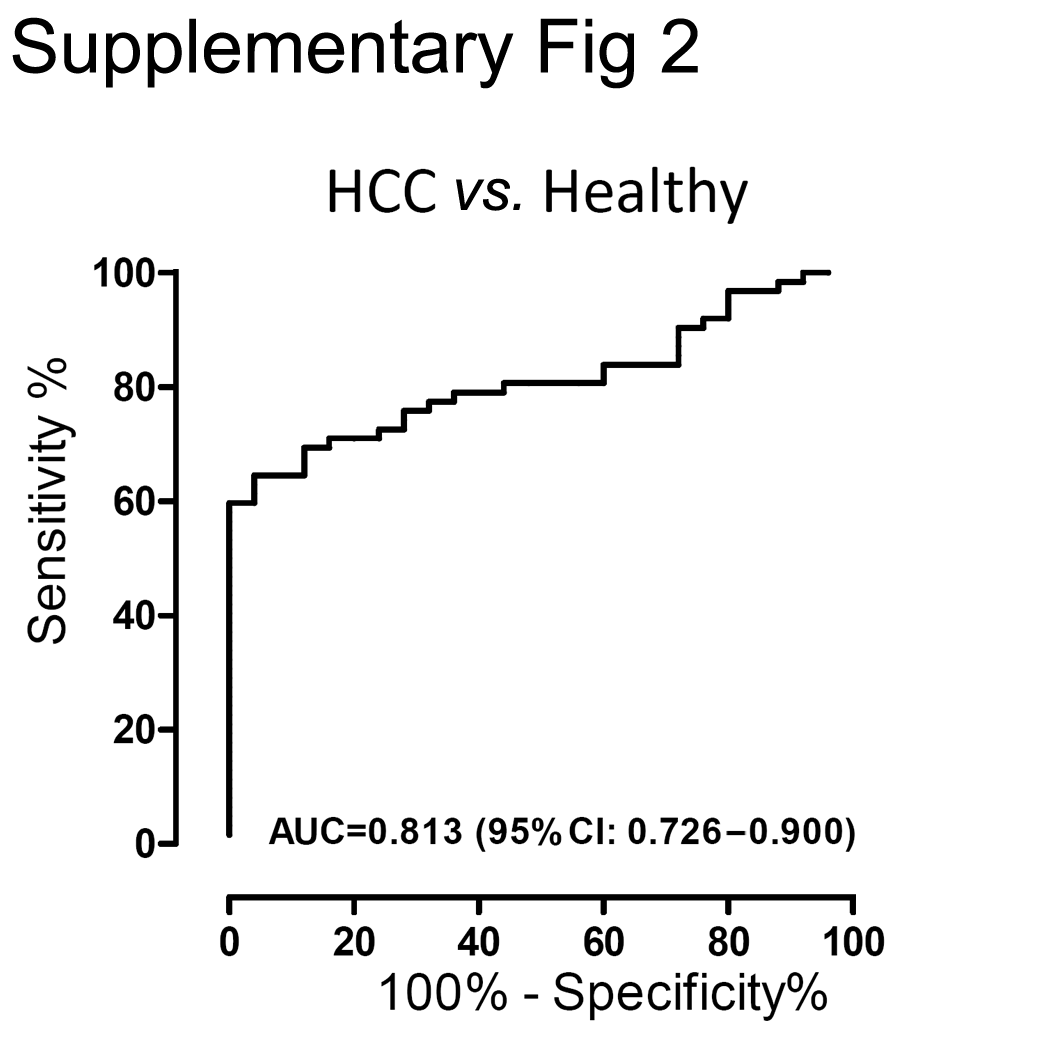

Supplement: Additional file 2: Figure S2 — ROC curve of AFP evaluating those with HCC (n = 62) and healthy controls (n = 25). [file 1479-5876-12-190-S2.tiff]
